# Molecular Characterisation of Bacterial Community Structure along the Intestinal Tract of Zebrafish (*Danio rerio*): A Pilot Study

**DOI:** 10.5402/2012/590385

**Published:** 2012-02-14

**Authors:** Chuan-Ching Lan, Donald R. Love

**Affiliations:** ^1^School of Biological Sciences, University of Auckland, Private Bag 92019, Auckland 1142, New Zealand; ^2^Labplus, Auckland City Hospital, P.O. Box 110031, Auckland Mail Centre, Auckland 1148, New Zealand

## Abstract

The bacterial composition along the intestinal tract of *Danio rerio* was investigated by cultivation-independent analysis of the 16S rRNA gene. Clone libraries were constructed for three compartments of the intestinal tract of individual fish. 566 individual clones were differentiated by amplified 16S rRNA gene restriction analysis (ARDRA), and clone representatives from each operational taxonomic unit (OTU) were sequenced. As reported in other studies, we found that *Proteobacteria* was the most prominent phylum among clone libraries from different fish. Data generated from this pilot study indicated some compositional differences in bacterial communities. Two dominant classes, *Gammaproteobacteria* and *Bacilli*, displayed different levels of abundance in different compartments; *Gammaproteobacteria* increased along the intestinal tract, while *Bacilli* decreased its abundance along the proximal-distal axis. Less obvious spatial patterns were observed for other classes. In general, bacterial diversity in the intestinal bulb was greater than that in the posterior intestine. Interindividual differences in bacterial diversity and composition were also noted in this study.

## 1. Introduction

Fish intestinal microflora comprises aerobic, facultative anaerobic, and obligate anaerobic bacteria. As found in humans, the microbial community may change with age, nutritional status, and environmental conditions [1]. Colonisation primarily takes place on the skin, in the gills, and in the intestine of fish. In the case of zebrafish, the active swallowing of water that occurs as early as 4 days of fertilisation (dpf) is the main route for microbial colonization of the intestine as bacteria are found in the mouth, pharynx, esophagus, and intestinal bulb [2]. From 4 dpf, bacterial abundance increases. Approximately 10^9^ 16S rRNA gene copies have been measured in the adult zebrafish digestive tract, indicating that the fish intestine provides favourable ecological niches for bacteria [2].

Extensive work on surveying intestinal microflora in freshwater fish has been carried out for decades, although most have relied on cultivation-based methods. However, these methods have been reported to recover only a fraction of the total bacterial community [3, 4]. This lack of recovery is due to unknown growth conditions, obligate requirements of coexisting bacterial species or host-produced factors, and the selective bias of media [5]. Cultivation-independent methods using total DNA extracted from intestinal samples have been applied to characterize the bacterial community in both marine and freshwater fish species [3, 6–16]. Several publications investigating bacterial microflora in different fish species show that the microflora predominantly consists of *Proteobacteria* species [2, 6, 8, 15, 17, 18].

The largest dataset of zebrafish microbiota reported to date has been described by Roeselers et al. [19]. The dataset consists of seven clone libraries, with a total of 3719 16S rRNA sequences, generated by Sanger sequencing, and 17,763 pyrosequencing reads from three libraries. The 16S rRNA sequences contained 370 phylotypes among Sanger sequencing libraries and 637 phylotypes among the pyrosequencing libraries (defined by 97% pairwise sequence identity), which were assigned to 13 phyla. The two principal phyla, *Proteobacteria* and *Fusobacteria*, represented 79.41% and 13.64%, respectively, of all bacterial clones. The remaining phyla comprised *Firmicutes*,* Bacteroidetes*,* Verrucomicrobia*,* TM7*,* Planctomycetes*,* Nitrospira*,* Deinococcus-Thermus*,* Tenericutes*,* Acidobacteria*, and *Cyanobacteria*. Only one phylum, *Proteobacteria*, is consistently found in all seven libraries. All except one library consist of members of the second most dominating phylum *Fusobacteria*.

To date, an investigation of the spatial bacterial diversity along the zebrafish intestinal tract has not been reported. In the study described here, culture-independent methods including amplified ribosomal DNA restriction analysis (ARDRA) and partial sequencing of the 16S rRNA gene were used to characterise the bacterial community of three intestinal compartments in zebrafish.

## 2. Materials and Methods

### 2.1. Zebrafish Husbandry and Intestinal Sample Collection

Adult zebrafish (all male, approximately 1 year old) were maintained in 2.5 L tanks in a high-density vertical rack system. The circulating water was filtered using a Millennium 2000 TM filtration system and was maintained at 26°C, with a conductivity of 500 to 600 *μ*S and pH of 7.3 to 8.0. Fish were fed dry food in the morning and *Artemia* in the afternoon. On the day of harvesting, no dry food was given in the morning. Three male fish housed in the same tank were euthanised with tricaine, and the fish were rinsed several times with autoclaved water. Each whole intestine was surgically removed in a sterile Petridish and divided into three sections: intestinal bulb (excluding esophagus) (IB), midintestine (MI), and posterior intestine (PI) according to the graphic drawing of the compartmentalised intestine described by Wallace et al. [20].

### 2.2. DNA Extraction from Intestinal Samples

The intestinal sections were placed in a bead-beating vial containing 0.4 mL of SDS lysis solution (10% SDS, 0.5 M Tris/HCl pH 8.0, 0.1 M NaCl) and 0.5 mL of 0.1 mm-diameter zirconia/silica beads (BioSpec). Bead beating was performed for 4 minutes at the maximum setting (4) using a FastPrep instrument (QBiogene, Irvine, CA). The supernatant was removed, and lysozyme was added to a final concentration of 10 mg/mL, followed by incubation at 42°C for 30 minutes. After the addition of ammonium acetate to a final concentration of 2.14 M, the solution was incubated at −20°C for 5 minutes and subsequently centrifuged at 13,000 g for 5 minutes. DNA was precipitated by adding of 0.7 volumes of room temperature isopropanol and was recovered by centrifugation at 13,000 g for 60 minutes at 4°C. The resulting DNA pellet was subjected to a final wash with 95% ethanol and resuspended in sterile DNase free water. 

### 2.3. 16S rRNA PCR, Library Construction, and ARDRA

16S rRNA PCR was performed using universal bacterial primers 27f (AGAGTTTGATCMTGGCTCAG) and 1472r (TACGGYTACCTTGTTACGACTT) [21]. Each 10 *μ*L reaction contained 1.28 mg/mL of BSA (Sigma-Aldrich), 1X i-StarTaq PCR Buffer (iNtRON Biotechnology), 200 nM of 27f forward primer, 200 nM of 1492r reverse primer, 0.25 mM of each dNTP (Invitrogen), 0.5 U i-StarTaq (iNtRON Biotechnology), and 50 to 200 ng of extracted DNA from each intestinal compartment. Reactions were incubated initially at 94°C for 10 min, followed by 30 cycles of 94°C for 1 min, 52°C for 1 min, and 72°C for 2 min, and a final extension at 72°C for 10 min. Negative control reactions were performed with sterile water. The products were resolved by 1% agarose gel electrophoresis. Ten independent 10 *μ*L PCRs for each compartment were pooled and purified using a MinElute PCR clean-up kit (Qiagen). The purified PCR product was ligated with the pGEM-T-Easy vector at a 3 : 1 (insert to vector) molar ratio. The ligation mixture was used to transform One Shot TOP10 chemically competent cells (Invitrogen), which were subsequently plated to L-plates containing ampicillin at 50 *μ*gmL^-l^and incubated overnight at 37°C. Ninety-six colonies were randomly picked and grown overnight at 37°C. The next day, glycerol stocks were made of each culture, which were stored at −80°C. Each culture was diluted 1 : 1 with sterile water and the cells were lysed by incubation at 95°C for five minutes. 1 *μ*L of each lysate was used in a 20 *μ*L colony PCR containing 1X PCR Buffer (Applied Biosystems), 2 mM MgCl_2_, 300 nM of SP6 and 300 nM of T7 primers, 0.1 mM of each dNTP (Invitrogen), and 0.5 U of AmpliTaq (Applied Biosystems). 1 *μ*L of each reaction was resolved in a 1% agarose gel to determine the lengths of the amplicons. The remaining 19 *μ*L of each PCR was incubated with 5U of *Hae*III (Invitrogen) at 37°C for 16 hours. 3 *μ*L of each digested product was subjected to electrophoresis in a 6% polyacrylamide gel.

### 2.4. Gel Analysis

A photograph of each polyacrylamide gel was processed by normalization and background subtraction with mathematical algorithms using GelCompar II software (Applied Maths NV). The dendrogram including patterns from all clones was constructed by cluster analysis using the unweighted pair group method with average linkages (UPGMA)/Dice coefficient of similarity. Each ARDRA pattern family was manually assigned by visual inspection and was defined as an operational taxonomic unit (OTU).

### 2.5. 16S rRNA Gene Sequencing

At least one representative of a given ARDRA pattern (OTU) was sequenced using either SP6 or T7 primers. DNA sequencing of the cloned 16r RNA gene product was performed using BigDye Terminator kit (Applied Biosystems) version 3 and an Applied Biosystems model 3100 capillary sequencer. The identity of the sequences was queried using the “SeqMatch” function of the Ribosomal Database Project (RDP II) website [22]. GenBank accession numbers for each OTU were assigned reference number JF261633-JF261695.

### 2.6. Richness, Diversity Indices, and Statistical Analysis

Rarefaction analysis was performed using software of Analytic Rarefaction 1.3 (http://www.uga.edu/strata/software/Software.html).

Library coverage was calculated by the formula of Good [23]  [1 − (*n*/*N*)] × 100, where “*n*” indicates the number of OTUs appearing only once in the library and “*N*” indicates the total number of clones examined. Chao [24] was calculated in the software application EstimateS (Version 8.2.0, http://viceroy.eeb.uconn.edu/estimates).

Two indices were used to assess diversity in each library [25]. The first was the (*H'*) = −∑*Pi*ln⁡⁡(*Pi*), in which *Pi* represents the proportion of clones with a given OTU compared to the total number of clones in the library. The second index was the Simpson reciprocal diversity index (1/D), which was calculated using the formula:  1/∑(*n*/*N*)^2^. Here, “*n*” represents the number of clones of a particular OTU and “*N*” represents the number of clones of all OTUs. Evenness index (*E*) was calculated using the formula:  *H*′/log⁡⁡*S* in which *H*′ is the Shannon-weaver index and *S* is the total number of OTUs in the library. The compositions of libraries were compared using the Sorensen index (Cs) = 2*j*/(*a* + *b*), in which *j* is the number of OTUs found in both samples A and B; a is the number of OTUs in sample A, and b is the number of OTUs in sample B. Values of richness, diversity indices, and relative bacterial composition at the class level were compared by use of repeated-measures ANOVA, followed by a Tukey multiple-comparison test.

## 3. Results

### 3.1. Sequence Analysis of the Clone Libraries

Nine 16S rRNA libraries were constructed that represented three intestinal compartments (IB, MI, and PI) of three adult male zebrafish. The final analyzed dataset comprised of 566 clones, represented by 63 unique OTUs. Clones that failed to yield good quality sequences were excluded from further analysis. The identity of each OTU that occurred in each library was confirmed by partial sequencing of the 16S rRNA gene.

Using the SeqMatch query of RDP II, bacterial sequences were assigned to a total of ten phyla. 10 of 13 phyla identified by Roeselers et al. [19] were also identified in the libraries presented here (Table 1). No library clones were assigned to *Bacteroidetes*. Most of clones in our collection belonged to *Proteobacteria *(mean ± SD = 59.20% ± 17.03%) and *Firmicutes* (23.81% ± 10.79%) (Table 1). *Proteobacteria*, which were represented by 29 OTUs, consisted of four classes: unclassified *Proteobacteria*, *Alphaproteobacteria*, *Betaproteobacteria*, and *Gammaproteobacteria*. The last class was the most prominent, harbouring the highest number of unique OTUs (13 OTUs). The second most abundant phylum in our collection was *Firmicutes*, consisting of only one class, *Bacilli*. Only the phyla *Proteobacteria*, *Firmicutes*, *Actinobacteria*, and *Fusobacteria* were found in all fish. No single OTU was consistently found in all compartments of all three fish.

### 3.2. Richness of Bacterial Communities in Different Compartments

**Figure 1:**
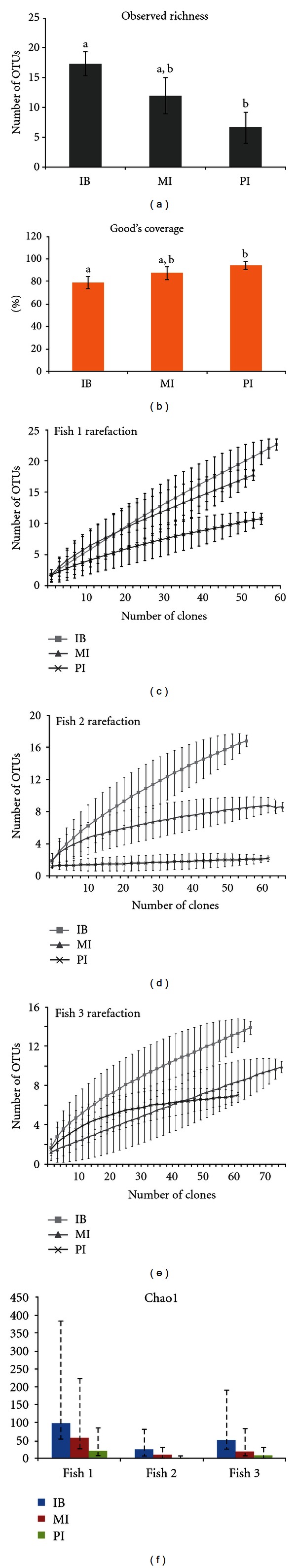
Richness analyses of the clone libraries: IB, MI, and PI. (a) represents the average number of OTUs observed in each compartment. (b) shows Good's coverage, which was used to calculate coverage for clone libraries; error bars indicate the SEM of three fish. (c), (d), and (e) show rarefaction analyses of the IB, MI, and PI libraries in Fish 1, Fish 2, and Fish 3, respectively; error bars indicate 95% confidence intervals. (f) shows the total number of OTUs as predicted by Chao for IB, MI, and PI libraries in each fish; error bars represent 95% confidence intervals. (a) and (b), values that are not sharing a common superscript letter are different at *P* values of < 0.01 and < 0.05, respectively.

The total richness (the total number of observed OTUs in each library) was found to be highest in the IB and lowest in the PI (*P* < 0.01) (Figure 1(a)). The Formula of Good [23] was used to assess library coverage. If a library has coverage of 99%, this means that one new OTU would be discovered for every 100 additional clones sampled. Analysis of the libraries revealed that the Good's coverage within the PI library was the highest (mean = 94%), followed by those in the MI library (87%), and in the IB library (79%). The difference was significant between the PI and IB libraries (*P* < 0.05) (Figure 1(b)). Rarefaction curves were constructed by plotting the number of observed OTUs against the number of clones (Figures 1(c), 1(d), and 1(e)). In Fish 1, the curves of all libraries (IB, MI, and PI) failed to reach an asymptote, which suggested that the libraries were not sampled to saturation and that further OTUs could be discovered [26]. In Fish 2, the MI and PI libraries were better sampled than the IB library. In Fish 3, the PI library was better sampled than the IB and MI libraries. Overall, the rarefaction curves also demonstrated that the sampled communities were less diverse in the PI than in the IB and MI libraries. The number of unseen OTUs is represented by the gap between the observed phylotypes and the number of OTUs estimated by Chao [24]. This comparison shows unseen OTUs in the IB libraries, whereas the unseen OTUs were lower for PI libraries (Figure 1(f)). Critically, the 95% confidence intervals (CIs) for the Chao in each intestinal compartment overlap (Figure 1(f)). Therefore, the null hypothesis that there is no difference between Chao values in the three compartments cannot be rejected at a significance level of 0.05.

### 3.3. Evenness and Diversity of Bacterial Communities in Different Compartments

**Figure 2:**
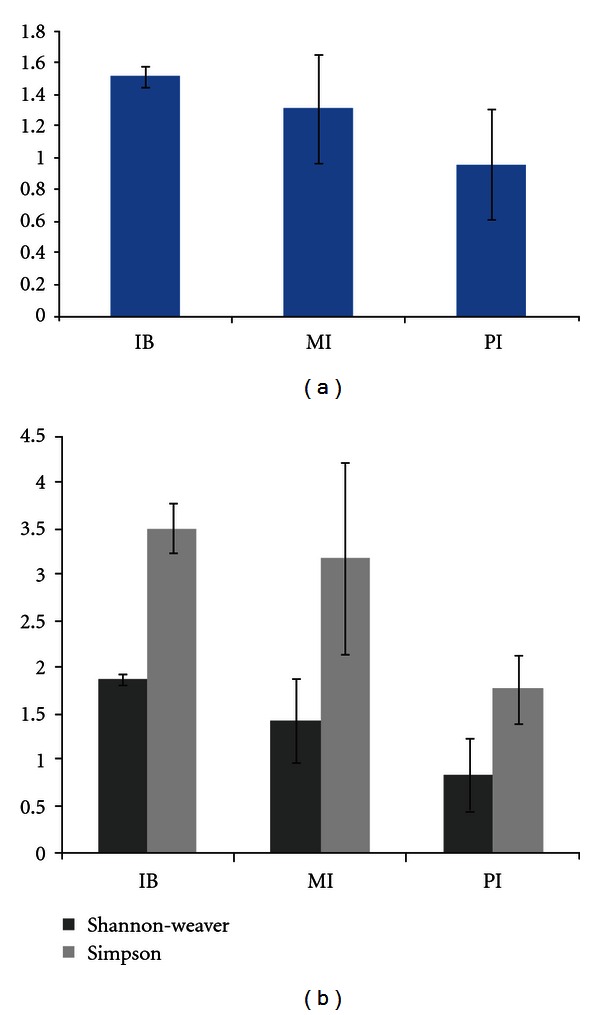
Evenness and diversity indices of clone libraries for IB, MI, and PI. The evenness was calculated for the clone libraries from the same compartment (a). Shannon-weaver (*H*′) and Simpson's reciprocal (1/D) were used for diversity estimation (b). Error bars indicate SEM of measurements from three fish. No significant difference was found for diversity indices and evenness between any two compartments.

The index of evenness (E), which is proportional to the number of clones that belong to each OTU, was higher in both IB (mean ± SEM = 1.53 ± 0.05) and MI (1.32 ± 0.33) than in PI (0.96 ± 0.35). The Shannon diversity index (*H*′), positively correlates with species richness and evenness, which means that the diversity of the library increases when a new additional species is discovered or species evenness is increased. The Shannon index revealed a similar trend to that shown by the E estimator (Figure 2(a)). The second index of diversity, the Simpson reciprocal diversity index (1/D), is a dominance measure, meaning that a community in which one or two species make up a large proportion of the community is considered less diverse than one in which several different species have similar abundance. Again, a similar trend was observed as described above (Figure 2(b)). The standard errors of mean for the MI and PI libraries for *E*, *H*′, and 1/D estimators were large; therefore, there appears to be no obvious difference in evenness and diversity between the libraries.

### 3.4. Similarities of Bacterial Communities in Different Compartments and Different Individuals

**Figure 3:**
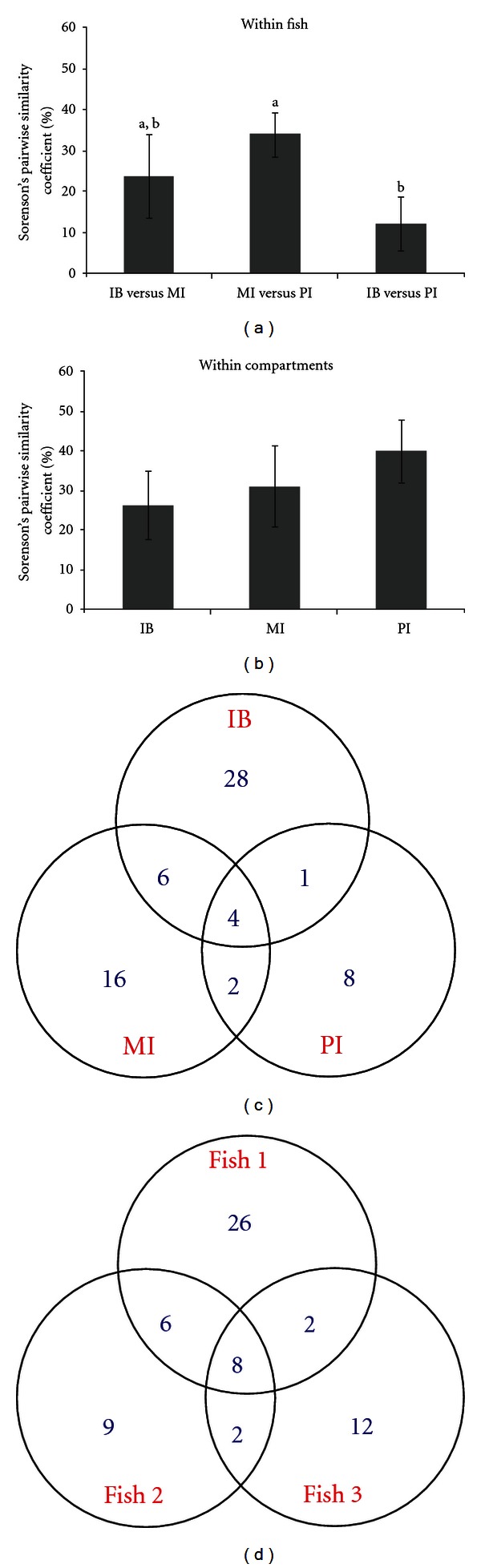
Sorenson's similarity index for “within fish” and “between-fish” comparisons. (a) shows the “within-fish” comparisons. Similarities were calculated based on pair-wise comparisons of any two compartments in the same fish; values that are not sharing a common superscript letter are different (*P* < 0.05). (b) shows the “between-fish” comparisons. Values were derived from the pair-wise comparison of the same compartment from any two fish. Error bars indicate SEM. The Venn diagram shows the distribution of all 65 OTUs identified in the three compartments IB, MI, and PI, revealing a shared community of 21 OTUs found in all three compartments (c). The second Venn diagram indicates the number of shared and unshared OTUs between the three collections, each of which consists of pooled clones from the same fish (d).

Sorenson's pairwise similarity coefficient was calculated to assess the similarity of bacterial communities, where 100% describes complete identity and 0% indicates no common OTU between two communities. Within individual fish, the similarities were generally low between any two compartments (11.8–30.4%), with 2 to 5 OTUs being shared between compartments. The lowest similarity (11.8%) was found between IB and PI libraries (Figure 3(a)). A significant difference was found between MI and PI, and IB and PI, comparisons (*P* < 0.05). The bacterial communities from the same compartment of different fish were also compared. The pair-wise comparisons showed similarities of 26.2% to 40.0%, which appear to be higher than those of  “within-fish” comparisons (Figure 3(b)). More OTUs were shared between IB and MI (10) than between IB and PI (5) (Figure 3(c)). When comparing shared OTUs between individuals, 8 OTUs were found in all three fish and between 10 to 14 OTUs were present in any of the two fish (Figure 3(d)).

### 3.5. Composition of Bacterial Communities along the Intestinal Tract

#### 3.5.1. *Actinobacteria*

**Figure 4:**
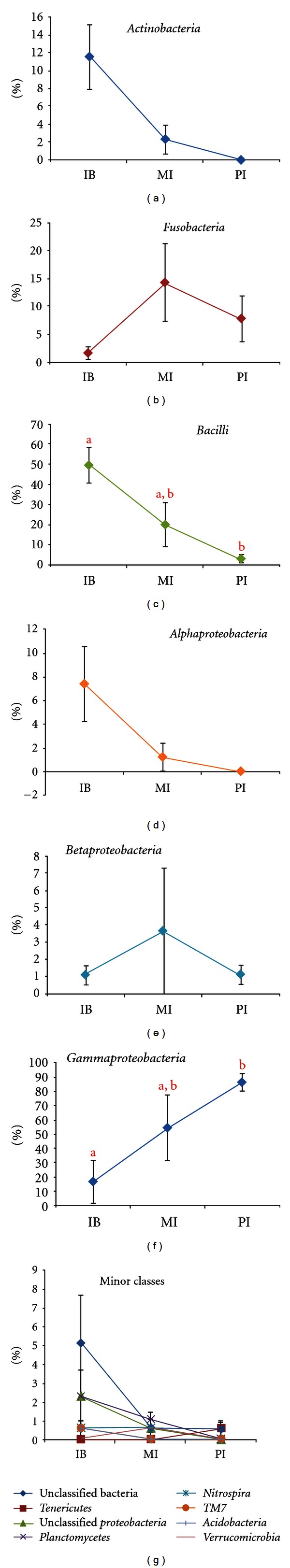
Distribution of bacterial classes in the clone libraries of IB, MI, and PI. The classes include *Actinobacteria *(a), *Fusobacteria *(b), *Bacilli *(c) *Alphaproteobacteria *(d), *Betaproteobacteria *(e), *Gammaproteobacteria *(f), and minor classes (g). Each of the classes in the “minor classes” group (except unclassified bacteria) constituted 0–2% of the clone libraries. Error bars indicate SEM of three fish. In *Bacilli *(c) and *Gammaproteobacteria *(f), values that are not sharing a common superscript letter are different (*P* < 0.05). (Note that the scale of the *y*-axis differs between panels.)

OTUs belonging to the class *Actinobacteria* were found to be most abundant in the IB (mean = 11.55%) and were absent in the PI in all fish. No comparison can be made with the PI, from which no *Actinobacteria* clones were recovered (Figure 4(a)). The *Actinobacteria *OTUs had highest similarity to members of the genera *Pseudonocardia*, *Kocuria*, *Mycobacterium*, *Dermacoccus*, *Propionibacterium*, *Microbacterium*, and *Brevibacterium*.

#### 3.5.2. *Fusobacteria*

Only two *Fusobacteria* clones were identified in the libraries, which displayed high levels of sequence similarity (96.4–99.1%) to members of the genus *Cetobacterium*. Large interindividual differences were observed. In the case of Fish 1, MI and PI exhibited similar levels of abundance (16.36% and 15.52%, resp.), but, in Fish 2, MI exhibited a relatively high abundance of 25% compared to IB and PI (3.51% and 1.56%, resp.). In contrast, between 1.30% and 6.35% clones belonged to *Fusobacteria *in Fish 3 (Figure 4(b)).

#### 3.5.3. *Bacilli*

The phylum *Firmicutes* was only represented by the *Bacilli* class in the libraries. The *Bacilli* abundance decreased along the intestinal tract (Figure 4(c)). The difference was most pronounced between the IB (mean = 49.76%) and PI (mean = 3.27%) (*P* < 0.05). One *Bacilli *clone that dominated the IB libraries had the closest match (99% identity) to* Bacillus cereus*, AL1 (AY129651).

#### 3.5.4. *Alphaproteobacteria* and *Betaproteobacteria*

The abundance of *Alphaproteobacteria *appeared to decrease from the IB to MI with complete absence in the PI (Figure 4(d)). *Betaproteobacteria *generally occurred at lower abundance (less than 1.8%) in all compartments, except for the MI (Figure 4(e)).

#### 3.5.5. *Gammaproteobacteria*

*Gammaproteobacteria *was the most abundant class in PI libraries (mean = 86.7%). The abundance in IB (mean = 16.6%) was significantly lower in the IB compared to the PI (*P* < 0.05) (Figure 4(f)). Although *Gammaproteobacteria *was represented by the highest number of unique OTUs (13), one OTU is the predominant *Gammaproteobacteria* member in the PI libraries, which displayed 99% sequence identity to *Vibrio cholerae* M66-2 (CP001233).

#### 3.5.6. Other Minor Classes

Other classes including *Tenericutes*, *Planctomycetes*, *Nitrospira*, *TM7*, *Acidobacteria*, and unclassified *Proteobacteria* were minor constituents in all libraries (0 to 2%) (Figure 4(g)). Higher proportions of unidentified bacteria were detected in the IB compared to the MI and PI.

## 4. Discussion

Most of the phyla identified in this study have been reported earlier, with the greatest number of clones affiliated with *Proteobacteria*, especially the *Gammaproteobacteria* class [19, 27, 28]. One prominent difference found in the present study compared to the study of Roeselers et al. [19] was that *Firmicutes *represented the second most abundant phylum. *Firmicutes* represents one of the most abundant phyla in the mammalian intestine, and members of *Clostridia* within *Firmicutes* are obligate anaerobes [29]. The zebrafish intestine is predicted to be more tolerant to oxygen than the mammalian intestine [27]. In our study, the *Firmicutes *was solely represented by an OTU closely related to *Bacillus cereus *(99% pair-wise identity), which is aerobic or facultatively anaerobic. *Bacilli* are the only *Firmicutes* members that are retained after the transplantation of mouse microbiota to germ-free zebrafish, which is likely to reflect their oxygen requirements. In addition, a 16S rRNA gene analysis of bacterial diversity in the mucus layer of the rainbow trout intestine (*Oncorhynchus mykiss*) also found *Firmicutes *to be the second most dominant phylum [13]. Overall, 10 out of 13 phyla reported by Roeselers et al. [19] were also present in our clone libraries, which indicates that the ARDRA method is capable of identifying most phyla, including the rare ones.

The critical aim of the study reported here was to discern spatial differences in bacterial diversity and composition along the intestinal tract and to determine if there were differences between zebrafish. When richness estimators including total richness, Good's coverage, rarefaction curves, and Chao1 were applied to estimate the bacterial diversity in different compartments, the IB seemed to harbour most diverse bacterial communities while PI libraries were much less diverse. Higher richness in the IB was mainly attributed to higher proportions of OTUs that only occur once (known as “singletons”). PCR artefacts may contribute to increased abundance of rare OTUs, leading to overestimation of richness in the IB libraries [30]. However, low coverage in these libraries means that more clones need to be sampled to reveal the true richness. Shannon-weaver and Simpson's reciprocal indices indicated that diversity varied in different compartments, but interindividual differences were observed but with no consistent trend with regard to spatial differences. In general, the PI libraries were less diverse than the other two libraries, as reflected by lower index values.

Pair-wise comparisons of similarity using Sorenson's method were made between any two compartments within the same fish (“within-fish” comparisons) or the same compartment from any two fish (“between-fish” comparisons). The “within fish” comparisons yielded generally low similarities; the comparison IB versus PI was most dissimilar, implying that each carried distinctive bacterial lineages. The “between-fish” comparisons showed slightly higher similarities than the “within-fish” comparisons, but there was still strong interindividual variability in the same compartment. Notably, the PI libraries from different fish were most similar compared to the other two, as they were dominated by bacteria that showed a high level of homology to *Vibrio cholerae* M66-2 (CP001233).

In this study, we evaluated bacterial composition at the class level in three compartments IB, MI, and PI. Of the classes examined, *Bacilli* and *Gammaproteobacteria* exhibited contrasting trends of distribution. The abundance of *Gammaproteobacteria* tended to increase along the proximal-distal axis, while *Bacilli* displayed the opposite trend. Interestingly, both classes were dominated by one OTU, instead of being represented by several OTUs. *Gammaproteobacteria* were predominantly represented by the clone with the highest sequence identity to *Vibrio *genus. Fish may benefit from *Vibrio *colonisation in the intestine; the family *Vibrionaceae *have been reported to exhibit chitinolytic ability [31, 32], which can in turn help fish to digest their prey such as *Artemia *spp. that are rich in chiton [33, 34]. Likewise, *Firmicutes* were largely represented by an OTU closely related to *Bacillus cereus *(99% pair-wise identity). Their preferential distribution in the IB is puzzling and may be specific to our facility as Roeselers et al. [19] found *Firmicutes *to be minor constituents in most of the clone libraries.

Our results suggest that each compartment may carry distinctive bacterial lineages, but the spatial patterns of composition and diversity are somewhat random in each fish. It should be stressed, however, that our limited sampling of each anatomic site militates against making a bold claim regarding regionally unique lineages. Further validation of our data could be undertaken using more fish and quantitative PCR with genus-specific primers or fluorescence *in situ* hybridization.

In farmed juvenile Atlantic salmon (*Salmo salar *L.), the TGGE profile of 16S rRNA products showed two major bands belonging to *Pseudomonas* spp., which were consistently found in all three compartments (stomach, pyloric caeca, and intestine), revealing a similar diversity along the intestinal tract [4]. The authors concluded that the examined compartments may lack regional specialisation to select its constituents. In a recent study, the adult zebrafish intestinal tract was further divided into seven roughly equal-length segments (S1 to S7): S1-S2 cover IB, S3-S4 cover MI, and S5–S7 cover PI [35]. Transcriptome analyses reveal that segments S1 to S5 have very similar expression patterns, especially genes with known function in the digestive tract. Segments S6 and S7 exhibit similar expression patterns, which are distinct from S1 to S5. It was concluded that segments S1 to S5 resemble human and mouse small intestine whereas S6 is analogous to human cecum and rectum and S7 to human rectum. If the first five segments (approximately corresponding to IB and MI compartments) are of similar function, then the local environments may be similar and not distinctive enough to result in unique bacterial communities. Due to functional and anatomical differences in IB and MI (in PI the intestinal folds are much shorter and are often covered with a thick layer of mucus [20]), they may exercise different selection pressure in the establishment of bacterial lineages. Our data appear to support this conclusion as richness, community composition (measured by Sorenson's pair-wise similarity), and the distribution of two classes of bacteria *Bacilli* and *Gammaproteobacteria* were found to be the most different between the IB and PI. In view of the relative small sample size (3 fish), these data require replication with a larger study population, and more clones need to be sampled. For other classes, the interindividual variability may prevent any clear pattern of species composition to be seen. This raises the question as to whether these communities are functionally the same and also if they are stable.

In this pilot study, we used ARDRA to determine the spatial heterogeneity of bacterial communities along the zebrafish intestinal tract. Some differences were noted, but, due to the limited number of sequenced clones, there is not enough power to unmask differences in composition or diversity along the tract. The use of deep sequencing on the variable regions of the 16S rRNA gene may present a more complete survey of bacterial taxa. Further understanding of the transition of bacterial community structure during zebrafish development, and the stability of the microflora within a defined spatial compartment, could be gained by these studies.

## Figures and Tables

**Table 1:** Comparison of bacterial phyla identified in the study by Roeselers et al. [19] and this study.

Phylum	Sanger sequencing clone libraries	Pyrosequencing libraries	This study^a^
*Proteobacteria*	79.41% ± 16.29%^b^	60.51% ± 35.39%^c^	59.20% ± 17.03%^d^
*Firmicutes*	1.01% ± 1.22%	6.36% ± 7.54%	23.81% ± 10.79%
*Bacteroidetes*	0.05% ± 0.12%	0.88% ± 0.80%	Not found
*Verrucomicrobia*	0.05% ± 0.14%	Not found	0.19% ± 0.34%
*Actinobacteria*	1.09% ± 2.39%	2.85% ± 4.68%	4.40% ± 1.24%
*TM7*	0.02% ± 0.05%	Not found	0.52% ± 0.49%
*Fusobacteria*	13.64% ± 14.35%	13.31% ± 20.82%	8.01% ± 4.41%
*Planctomycetes*	1.52% ± 2.53%	0.06% ± 0.10%	1.05% ± 0.51%
*Nitrospira*	0.03% ± 0.09%	Not found	0.39% ± 0.68%
*Deinococcus-Thermus*	Not found	0.33% ± 0.55%	Not found
*Tenericutes*	Not found	Not found	0.19% ± 0.34%
*Acidobacteria*	0.04% ± 0.09%	Not found	0.19% ± 0.34%
*Cyanobacteria*	2.71% ± 7.16%	15.14% ± 25.53%	Not found
Unclassified bacteria	0.39% ± 0.54%	0.54% ± 0.76%	2.04% ± 1.78%

^a^In this study, % of each phylum in the clone libraries (IB, MI, and PI) was derived by pooling clones from different compartments from each fish.^b^Mean ± SD of clone libraries (*n* = 7). The seven libraries include D.rerio.UNC.1, D.rerio.India.1, D.rerio.ZIRC.1, D.rerio.UO.1, D.rerio.WU.1, D.rerio.WU.2, and D.rerio.UW.1.^c^Mean ± SD of libraries (*n* = 3). The three libraries include D.rerio.India.1, D.rerio.UNC.1, and D.rerio.UW.1.^d^Mean ± SD of pooled clone libraries (*n* = 3).
